# Evolution of allostery in the cyclic nucleotide binding module

**DOI:** 10.1186/gb-2007-8-12-r264

**Published:** 2007-12-12

**Authors:** Natarajan Kannan, Jian Wu, Ganesh S Anand, Shibu Yooseph, Andrew F Neuwald, J Craig Venter, Susan S Taylor

**Affiliations:** 1 Department of Chemistry and Biochemistry, University of California, Gilman Drive, La Jolla, California, 92093-0654, USA; 2 Department of Biological Sciences, Science Drive 4, National University of Singapore, Singapore 117543; 3 J Craig Venter Institute, Medical Center Drive, Rockville, MD 20850, USA; 4 Institute for Genome Sciences and Department of Biochemistry and Molecular Biology, University of Maryland School of Medicine, HSF-II, Penn Street, Baltimore, MD 21201, USA; 5 Department of Chemistry and Biochemistry, and HHMI, University of California, Gilman Drive, La Jolla, California, 92093-0654, USA

## Abstract

**Background:**

The cyclic nucleotide binding (CNB) domain regulates signaling pathways in both eukaryotes and prokaryotes. In this study, we analyze the evolutionary information embedded in genomic sequences to explore the diversity of signaling through the CNB domain and also how the CNB domain elicits a cellular response upon binding to cAMP.

**Results:**

Identification and classification of CNB domains in Global Ocean Sampling and other protein sequences reveals that they typically are fused to a wide variety of functional domains. CNB domains have undergone major sequence variation during evolution. In particular, the sequence motif that anchors the cAMP phosphate (termed the PBC motif) is strikingly different in some families. This variation may contribute to ligand specificity inasmuch as members of the prokaryotic cooA family, for example, harbor a CNB domain that contains a non-canonical PBC motif and that binds a heme ligand in the cAMP binding pocket. Statistical comparison of the functional constraints imposed on the canonical and non-canonical PBC containing sequences reveals that a key arginine, which coordinates with the cAMP phosphate, has co-evolved with a glycine in a distal β2-β3 loop that allosterically couples cAMP binding to distal regulatory sites.

**Conclusion:**

Our analysis suggests that CNB domains have evolved as a scaffold to sense a wide variety of second messenger signals. Based on sequence, structural and biochemical data, we propose a mechanism for allosteric regulation by CNB domains.

## Abstract 2

Analysis of cyclic nucleotide binding (CNB) domains shows that they have evolved to sense a wide variety of second messenger signals; a mechanism for allosteric regulation by CNB domains is proposed.

## Background

The cyclic nucleotide binding (CNB) domain is a conserved signaling module that has evolved to respond to second messenger signals such as cAMP and cGMP [1,2]. The CNB domain is ubiquitous in eukaryotes and controls a variety of cellular functions in a cAMP/cGMP dependent manner. Some of the well characterized CNB domain containing families in eukaryotes include: the protein kinase A (PKA) regulatory subunit that regulates the activity of PKA [3,4]; the guanine nucleotide exchange factor that regulates nucleotide exchange in small GTPases [5]; and the ion channels that regulate metal ion gating (reviewed in [6]).

CNB domains also occur in prokaryotes. The first characterized family containing a CNB domain in prokaryotes is the CAP (catabolite gene activator protein) family of transcriptional regulators [7] that contain a DNA binding helix-turn-helix (HTH) domain covalently linked to the CNB domain [8]. This domain organization is important for CAP function as it couples cAMP binding functions of the CNB domain with DNA binding functions of the HTH domain [9]. The CAP family is functionally diverse and, in addition to cAMP, responds to other exogenous signals, such as carbon monoxide (CO) and nitric oxide (NO) (reviewed in [10]). The cooA subfamily, for instance, responds to CO signals and binds a heme ligand in the cAMP binding pocket [11]. Likewise, the CprK subfamily of transcriptional regulators binds to ortho-chlorophenolic compounds in the cAMP binding pocket [12].

Crystal structures of CNB domains from both eukaryotes and prokaryotes have been determined and their structural comparison reveals a conserved mode of cAMP recognition [1] and regulation (reviewed in [13]). CNB domains are characterized by an eight stranded beta barrel domain (beta subdomain) [14] that is conserved among all CNB domain containing proteins [1]. A key structural region within the beta subdomain is the phosphate binding cassette (PBC) that anchors the phosphate group of cAMP [15]. CNB domains also contain a helical subdomain (henceforth called alpha subdomain), which, unlike the beta subdomain, is more variable in sequence and structure. The helical subdomain is also a docking site for the catalytic subunit of PKA [16].

An emerging theme in CNB domain signaling is the allosteric control of CNB domain functions. In the PKA regulatory subunit, for instance, cAMP binding to the beta subdomain causes conformational changes in the distal alpha subdomain, thereby releasing its inhibitory interactions with the catalytic subunit [17]. This propagation of the cAMP signal to distal regulatory sites was suggested to involve specific regions in the beta subdomain [18]. Specifically, a loop connecting the β2 and β3 strands (β2-β3 loop) was shown to undergo large chemical shift changes upon binding to cAMP [18]. While these and other studies have provided important insights into PKA allostery, it is not known whether this mode of regulation is unique to the PKA regulatory subunit or is conserved among other members of the CNB domain superfamily. Here, we address this question by extracting and analyzing the evolutionary information encoded within CNB domain containing sequences. Towards this end, we have identified nearly 7,700 CNB domain containing proteins, and classified them into 30 distinct families. A systematic comparison of these families reveals that the CNB domains recombine with a wide variety of functional domains to respond to diverse cellular signals. Statistical comparison of the evolutionary constraints imposed on CNB domain sequences reveals that the residues that anchor the phosphate group of cAMP (within the beta subdomain) have co-evolved with residues in the β2-β3 loop. Analyzing these residues in light of existing structural and biochemical data provides a model of allostery that is conserved through evolution.

In the following sections, we first describe the identification and classification of CNB domains to illustrate the diversity of this protein family, and later show how a comparative analysis of CNB domain sequences has provided insights into the evolution of allostery.

## Results and discussion

### Identification and classification of CNB domains in the public and Global Ocean Sampling data

Cyclic nucleotide binding domains in the National Center for Biotechnology Information's non-redundant amino acid database (NR) and Global Ocean Sampling (GOS) [19,20] data were identified using a combination of psi-blast profiles and motif models (see Materials and methods). This resulted in nearly 5,241 significant hits in NR and 2,455 hits in the GOS data. Most of the identified sequences were multi-domain proteins in that they contained other functional domains covalently linked to the CNB domain. Because these functional domains play an important role in CNB domain functions, they were used as markers for annotation and classification (see below).

**Figure 1:**
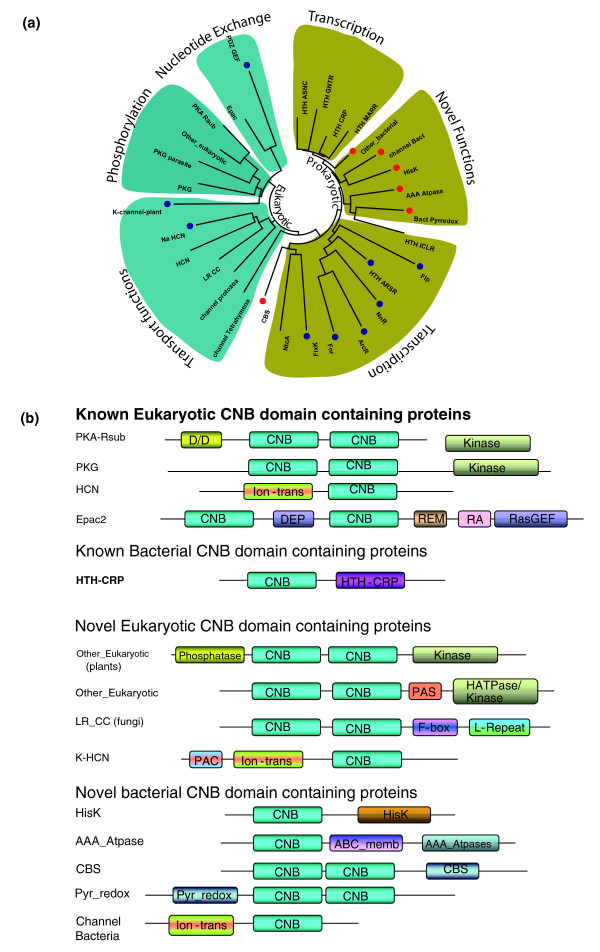
Classification and domain organization of CNB domain containing families. **(a) **Phylogenetic tree of the 30 identified families. Eukaryotic branches are shown in dark teal, while the prokaryotic branches are shaded in gold. Novel families in bacteria are indicated by red dots. Families that have a non-canonical PBC are indicated by blue dots. **(b) **Domain organization of known and novel CNB domain containing proteins in eukaryotes and prokaryotes.

The 7,696 CNB domain containing sequences can be classified into 30 distinct families (Figure 1) based on the sequence similarity within the CNB domain (see Materials and methods). These 30 families are predominantly eukaryotic or bacterial in origin (Table 1). The only significant hit in Archea was to a hypothetical protein (gi: 11498576) from *Archaeoglobus fulgidus*. CNB domains in eukaryotes can be broadly classified into five major categories: the kinase domain associated PKA and PKG families; the guaninine nucleotide exchange factor (Epac's); transmembrane domain containing HCN and Na channels; HCN type channels in protozoans; and CNB domains in metazoans and plants that are fused to functional domains such as PAS domains, PP2C like phosphatases and phospholipases ('Other_Eukaryotic' in Table 1). Several of these families/subfamilies are lineage-specific and contain domain combinations that have not been reported before. The PP2C like phosphatase, for instance, is a plant specific subfamily that contains a kinase domain carboxy-terminal of the CNB domain. The co-occurrence of kinases, phosphatase and CNB domains in the same operon is interesting because previous bioinformatics analysis had failed to provide any evidence for a cAMP or cGMP dependent regulation of kinase activity in plants [21].

**Table 1:** Classification of CNB domains in the public and GOS data

No.	Family name	NR/GOS count	Taxonomic origin	PBC consensus motif	Description
1	PKA-Rsub	301/0	Eukaryote	GELALIYGTP**R**AATVVA	cAMP dependent regulatory subunit that activates PKA
2	PKG	388/9	Eukaryote	GELALLYNDP**R**TATVIA	cGMP activated proteins that are typically attached to a kinase domain
3	PKG-parasites	362/11	Eukaryote	GERALLYDEP**R**SATIKA	A distinct group of PKGs in parasites that are also attached to kinase domains
4	Other_eukaryotic	940/201	Eukaryote	GELALLYNAP**R**AATVVA	CNB domains from metazoans and plants. These are attached to various functional domains such as PKs, PAS domains, PP2C like phosphatases and phospholipases
5	Epac	150/1	Eukaryote	GQLALVNDAP**R**AATIVL	cAMP-dependent guanine nucleotide exchange factors. Typically attached to an amino-terminal DEP domain and a carboxy-terminal RasGEF domain
6	PDZ-GEF	125/0	Eukaryote	GVSPTMDKEYMKGVMRT	A distinct class of Epac's, also called Epac6, which contains a PDZ domain in between the CNB and RasGEF domain. Epac's of this class contain a non-canonical PBC
7	K-channel	86/0	Eukaryote	GEVGVLCYRPQLFTVRT	Potassium channels specific to plants. Most of them contain an Ankryin repeat carboxy-terminal to the CNB domain
8	LR_CC	148/4	Eukaryote	GEIGVLLDPP**R**TATVRA	CNB domains found in metazoans and fungi, usually occur in tandem like the PKA regulatory subunit and contain a carboxy-terminal F-box domain and leucine rich domain
9	HCN	165/5	Eukaryote	GEICLLTRGR**R**TASVRA	cGMP-gated cation channels. Mostly present in metazoans
10	K_HCN	185/0	Eukaryote	GENFWLYGTKSNADVRA	Potassium channels that contain a PAC motif (motif carboxy-terminal of PAS) amino-terminal of the trans-membrane segment. This subfamily also contains a non-canonical PBC
11	Channel_Tetrahym.	218/44	Eukaryote	GEEDFFSGQP**R**TFTAKC	Likely HCN channels from the single celled eukaryote *Tetrahymena thermophila*. This subfamily is quite distinct from the HCN channels in higher eukaryotes
12	Channel_protozoa	587/41	Eukaryote	GEISFFTGLP**R**TASARS	Other HCN channels in protozoans
13	Bact_Pyrredox	38/70	Prokaryote	GEMGLISGRR**R**GATVRA	Tandem CNB domains that are attached to an amino-terminal pyridine nucleotide-disulphide oxidoreductase domain
14	Channel_Bact	99/79	Prokaryote	GEIALLTGGP**R**TATVRA	Bacterial CNBs that are attached to mechanosensitive ion channels
15	HisK	56/11	Prokaryote	GELSLLTGGP**R**SATVRA	Bacterial CNBs that contain a HisK like ATPase, carboxy-terminal of the CNB domain
16	AAA_Atpase	65/24	Prokaryote	GEMALLSGQE**R**KASVIA	A distinct sub-group containing AAA-ATPase domains attached to the CNB domain. Several members of this group contain an ABC-transporter like transmembrane region. The PBC arginine (Arg209) is quite variable within this family
17	NtcA	108/104	Prokaryote	GVLSLLTGSD**R**FYHAVA	Nitrogen responsive regulatory protein that contains a DNA binding domain (HTH) carboxy-terminal of the CNB domain
18	FixK	43/0	Prokaryote	G-ASLGGDHLFTAEA	Involved in nitrogen fixation and contains a HTH motif
19	FnR	176/53	Prokaryote	GEFDAIGSGHHPSFAQA	Transcriptional regulators that are implicated in oxygen sensing
20	ArcR	29/0	Prokaryote	PYGGLFTDDYYHESATA	Transcriptional regulator that is implicated in the aerobic arginase reaction. Arginine is used as a source of energy in bacteria
21	NnR	28/0	Prokaryote	GFARALQRGDYPGTATA	Transcriptional regulators that act on the *nir *and *nor *operons to achieve expression under aerobic conditions
22	CBS	173/51	Prokaryote	GERALLAGGPYSLTARA	This group contains tandem CBS domain located carboxy-terminal of the CNB domain
23	Other_bacterial	1553/1486	Prokaryote	GEMALLDGEP**R**SATVVA	Bacterial CNB domains that are attached to various functional domains such as CheY response regulators, Rhodanese homology domain, kinases and DNA binding domains
24	HTH_ICLR	33/14	Prokaryote	GEGAAFSEEP**R**STTVVA	Transcriptional regulator that is implicated in the repression of the acetate operon (also known as glyoxylate bypass operon) in *Escherichia coli *and *Salmonella typhimurium*
25	HTH_GNTR	85/52	Prokaryote	GEASLFDGEP**R**SATVVA	Transcriptional regulator containing a HTH domain and implicated in the repression of the gluconate operon
26	Flp	19/0	Prokaryote	GEEALFGESNHANYCEA	Involved in the bacterial oxidative stress response
27	HTH_ARSR	66/15	Prokaryote	GEAALFSNGPYPATAIA	Functions as a transcriptional repressor of an arsenic resistance operon. Dissociates from DNA in the presence of the metal
28	HTH_CRP	858/347	Prokaryote	GEAALFDGGP**R**PATAVA	Transcriptional regulation of the *crp *operon
29	HTH_MARR	143/20	Prokaryote	GEMALLDGGP**R**SADAVA	Repressor of genes that activate the multiple antibiotic resistance and oxidative stress regulons
30	HTH_ASNC	73/24	Prokaryote	GEIALLDGGP**R**SATATA	An autogenously regulated activator of asparagine synthetase A transcription in *Escherichia coli*

CNB domains are also prevalent in prokaryotes and some of the major groups include: the CRP family members (Marr, Arsr, AsnC, ICLR, GNTR) that contain a DNA binding domain covalently linked to the CNB domain; and a distinct class of DNA binding domain containing proteins (NnR, ArcR, Fnr and FixK) that are activated by second messenger signals such as NO, oxygen and heme [10]. In addition, our analysis reveals several novel families (CBS, HisK and AAA ATPases) in prokaryotes that lack the DNA binding domain, but conserve other functional domains (Table 1) such as histidine kinases (HisKs), cystathionine beta synthase (CBS) domains and AAA ATPases (AAA_Atpases in Table 1).

#### Expansion of transcriptional regulators in the Global Ocean Sampling data

Most of the GOS sequences, as expected, are prokaryotic in origin since they belong to families that are exclusively prokaryotic (Table 1). In particular, the CAP/CRP family, which contains a DNA binding domain covalently linked to the CNB domain and is implicated in the transcriptional regulation of genes, is greatly expanded in the GOS data (Table 1). The expansion of this family in the GOS data suggests that transcriptional regulation of many genes in oceanic microorganisms may be controlled in a cAMP or cGMP dependent manner. Also, the diversity displayed by the GOS sequences in the CAP family suggests that this family may regulate a wide variety of operons, in addition to the well studied *lac *operon [22]. In addition to the CAP family, the NtcA family (Table 1), which is involved in nitrogen fixing in cyanobacteria [23], is also expanded in the GOS data. More than half the GOS sequences fall into the 'Other_Bacterial' family (table 1), which is poorly characterized. This family is highly diverse and contains several distinct sub-families that are associated with functional domains such as Rhodanases, Chey response regulators and DUF domains (Table 1). Thus, GOS data greatly contribute to the diversity of the CNB superfamily and enable the use of statistical methods to understand how sequence divergence contributes to functional divergence (see below).

### Diversity in prokaryotes

Until now, the primary function of CNB domains in prokaryotes was believed to be in the transcriptional regulation of genes. However, our analysis suggests that other cellular processes, such as ATP production, protein phosphorylation and NADH production, may also involve CNB domain functions (Table 1). Of particular interest is the CBS domain associated CNB domains. CBS domains are known to function as sensors of cellular energy levels in eukaryotes as they are activated by AMP and inhibited by ATP. They are also implicated in various hereditary diseases in humans [24]. The function of CBS domains in prokaryotes, however, is poorly understood, although the crystal structure of a CBS domain from *Thermotoga maritime *has been determined as part of the structural genomics initiative [25]. The occurrence of both a CBS domain and a CNB domain in the same open reading frame suggests that, in some bacteria, ATP levels may be regulated in a cAMP-dependent manner. Structurally characterizing the full-length protein (CBS + CNB domain) may shed light on this regulatory mechanism in prokaryotes.

Other novel domains in prokaryotes that are fused to CNB domains include the HisKs that are involved in bacterial two component signaling, and the AAA class of ATPases (AAA_Atpases in Table 1) that control a wide variety of cellular functions in both eukaryotes and prokaryotes [26].

### A conserved core shared by the entire superfamily

**Figure 2:**
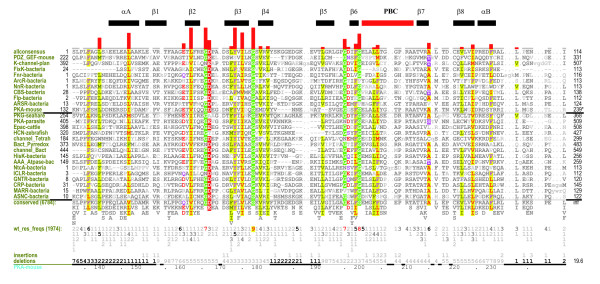
Conserved features of the CNB domain. A contrast hierarchical alignment showing conserved residues/motifs shared by the entire superfamily. The histograms above the alignments plot the strength of the selective constraints imposed at each position. Secondary structure is indicated directly above the aligned sequences with β-strands indicated by their number designations (that is, 1-7 correspond to the β1-β7 strands, respectively) and helices by their letter designations. The leftmost column of each alignment shows the sequences used in the display alignment. See Materials and methods for sequence identifiers. The background alignment of all CNB domain containing sequences are shown indirectly via the consensus patterns and corresponding weighted residue frequencies ('wt_res_freqs') below the display alignment. (Such sequence weighting adjusts for overrepresented families in the alignment.) The residue frequencies are indicated in integer tenths where, for example, a '5' indicates that the corresponding residue directly above it occurs in 50-60% of the weighted sequences. Biochemically similar residues are colored similarly with the intensity of the highlighting proportional to how strikingly foreground residues contrast with background residues.

**Figure 3:**
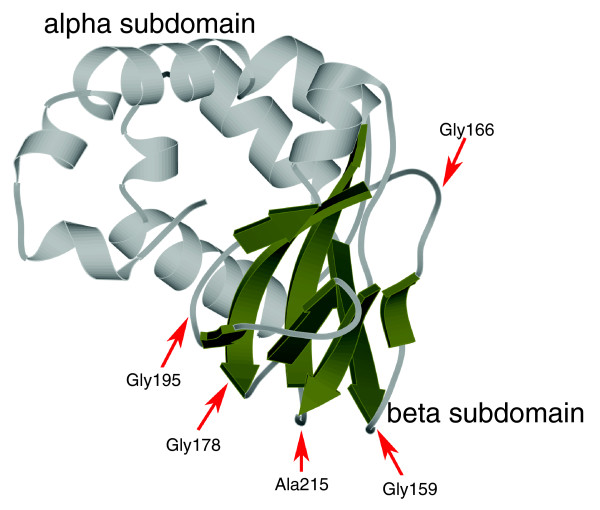
The structural location of the conserved glycines in the PKA regulatory subunit R1alpha (PDB: 1RGS). The alpha subdomain is shown in light gray and the beta subdomain is shown in dark grey. The glycines are shown in spheres representation.

While the functional domain linked to the CNB domain is unique to a given family or subfamily, the CNB domain is shared by the entire superfamily. A multiple alignment of nearly 7,000 CNB domain sequences (Figure 2) reveals key sequence motifs that are shared by the entire superfamily (Figure 2). These residues/motifs define the core of the CNB domain. Several of these core residues correspond to glycines (Gly159, Gly166, Gly178, Gly195, and Gly199) that are located in loops connecting the beta strands of the beta subdomain (Figure 3). Note that the residue numbers correspond to PKA-mouse numbering in Figure 2. The most conserved of these glycines is Gly178, which is located in the β3-β4 loop and adopts a main-chain conformation (phi = 85.0; psi = -176.5) that is disallowed for other amino acids in the Ramachandran map. The role of Gly178 is not obvious from crystal structure analysis; however, the remarkable conservation of this residue across diverse eukaryotic and prokaryotic phyla suggests an important role in CNB domain structure and function.

**Figure 4:**
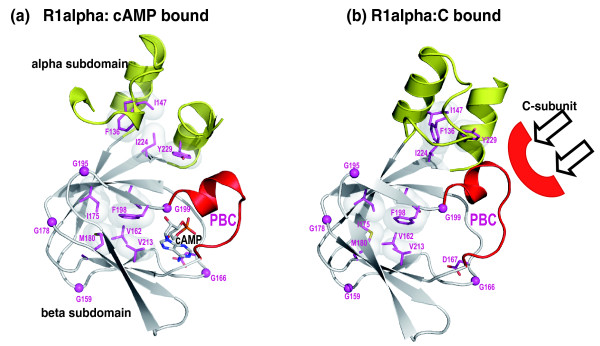
Core conserved residues shared by the entire superfamily and the conformational changes associated with the helical subdomain. **(a) **cAMP bound structure of the PKA regulatory subunit R1alpha (PDB: 1RGS). **(b) **Catalytic subunit (C-subunit) bound structure of R1alpha (PDB: 2QCS). The alpha subdomain is shown in yellow and the beta subdomain is shown in white. The PBC region is colored in red. The hydrophobic residues are shown in sticks and surface representation, and the glycine residues are shown in CPK representation. The core conserved residues are colored in gold.

In addition to the conserved glycines, CNB domains also conserve a hydrophobic core in the alpha and beta subdomains. The hydrophobic core in the alpha subdomain is formed by residues Phe136, Ile147, Tyr229, and Ile224, while the core in the beta subdomain is formed by residues Ile175, Met180, Val213, Val162, Phe198 and Tyr173 (Figures 2 and 4a). Comparison of the cAMP-bound and the catalytic subunit-bound structures of the PKA regulatory subunit (R1alpha) reveals that while the hydrophobic core in the beta subdomain is relatively stable in the two functional states, the hydrophobic core in the alpha subdomain is malleable and undergoes a conformational change upon binding to the catalytic subunit (Figure 4b). In particular, Tyr229, which packs up against the PBC in the cAMP-bound structure moves away from the PBC upon binding to the catalytic subunit (Figure 4b). Likewise, Phe136, which typically points away from the PBC, moves closer toward the PBC upon binding to the catalytic subunit. These coordinated changes in the helical subdomain were recently proposed to function as a latch for gating cAMP [13] and also shield cAMP from solvent. The conservation of these core residues across diverse families suggests that the conformational changes in the alpha subdomain may be a fundamental feature of all CNB domain functions.

### Functional diversity of the CNB module: a common scaffold to sense diverse ligands

**Figure 5:**
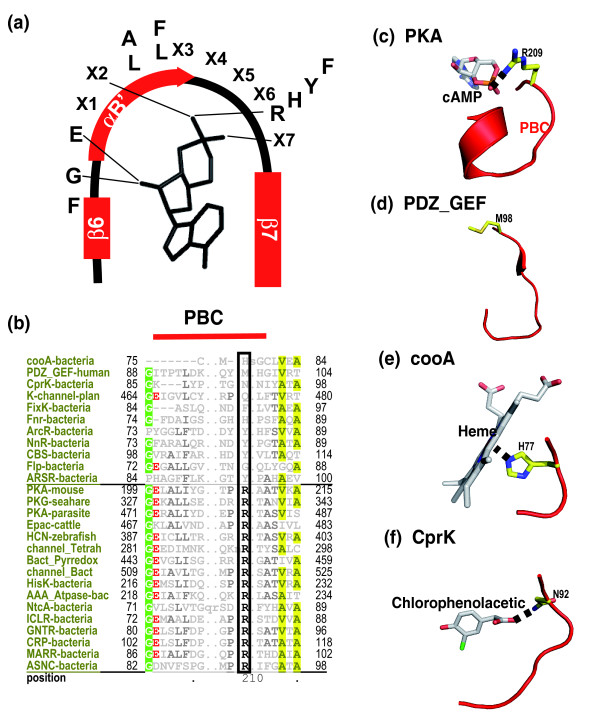
Sequence variation within the PBC and ligand specificity. **(a) **A schematic representation of the PBC showing the secondary structures and the consensus motif. **(b) **Families that contain a canonical and non-canonical PBC motif. Sequence alignment of the PBC region showing conserved and variable positions. Conserved residues are highlighted and Arg209 position is indicated by a black box. **(c-f) **The conformation of the PBC region in: the PKA regulatory subunit (PDB: 1RGS) (c); PDZ_GEF (PDB: 2D93) (d); cooA (PDB: 1FT9) (e); CprK (PDB: 2H6B) (f).

Having delineated the core residues/motifs of the CNB superfamily, we focused on motifs that contribute to the functional specificity of individual families. In particular, we focused on the PBC region (Figure 5a), which displays a strikingly different pattern of conservation in some families (Figure 5b). The canonical sequence motif in the PBC region is the FGE [L,I,V]AL [LIMV]X [PV]R^209 ^[ANQV] motif, where X is any amino acid. A key residue within this motif is a conserved arginine (Arg209), which coordinates with the phosphate group of cAMP (Figure 5c). While mutation of this arginine to a lysine in PKA reduces the affinity for cAMP by nearly ten-fold [27], some eukaryotic families, such as PDZ_GEF (PDZ domain associated family closely related to Epac), naturally contain a methionine or histidine at the Arg209 position (Figure 5b). Although the functional implications of this variation in PDZ_GEF (Figure 5d) are currently unclear, it is likely that this may alter the affinity for cAMP or facilitate binding of a different small molecule ligand. Notably, in the crystal structure of PDZ_GEF, which was solved as part of the RIKEN structural genomics initiative, the region analogous to the PBC region in PKA adopts a strikingly different conformation (Figure 5d) and is not bound to any ligand.

#### Sequence variation within the PBC region contributes to ligand specificity

Several families in prokaryotes conserve a non-canonical PBC motif. Some of these include the transcriptional regulators FixK, FnR, ArcR, NnR and ARSR (Figure 5b). Within the FixK, or cooA family, for instance, the observed sequence variation within the PBC region appears to contribute to ligand specificity inasmuch as the cooA family binds to a heme ligand in the cAMP binding pocket (Figure 5e). In the crystal structure of cooA, a conserved histidine, which occupies a position that is structurally analogous to Arg209 in PKA, coordinates with the heme and plays a key role in cooA activation [11]. Likewise, in the crystal structure of the transcriptional regulator CrpK bound to chlorophenolacetic acid [12], a structurally analogous asparagine (Asn92) residue hydrogen bonds to chlorophenolacetic acid (Figure 5f).

### Evolution of allostery in the CNB module

The ability of the CNB domain to bind to diverse ligands raises an important question: what features distinguish the cAMP binding families (ones that conserve a canonical PBC motif) from those that bind to other ligands? In order to address this question we used the CHAIN (Contrast Hierarchical Alignment and Interaction Network analysis) program, which quantifies the differences between two functionally divergent groups of sequences using statistical methods [28]. Using this program, we identified sequence features that distinguish the canonical PBC motif containing CNB domains from those that lack the canonical PBC motif. Analyzing these features in light of existing structural and biochemical data provides a model for allosteric regulation, which is likely conserved in all cAMP binding modules.

#### Selective constraints distinguishing the canonical PBC containing sequences

**Figure 6:**
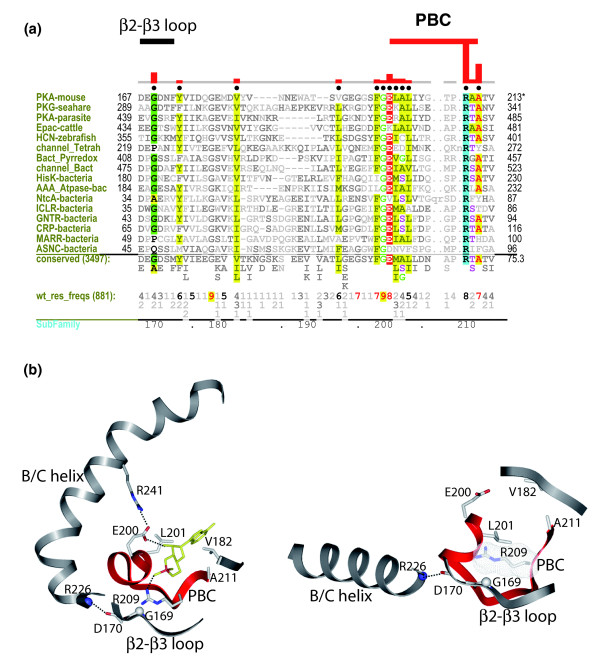
Sequence features that distinguish the canonical and non-canonical PBC containing sequences. **(a) **A contrast hierarchical alignment (see Figure 2 legend) showing residues (indicated by black dots above alignment) that distinguish the canonical PBC containing sequences from the non-canonical ones. Biochemically similar residues are colored similarly with the intensity of the highlighting proportional to how strikingly foreground residues contrast with background residues. **(b) **The allosteric link between the PBC and β2-β3 loop is shown using the cAMP bound and cAMP-free structures of the PKA regulatory subunit.

The key residues that distinguish the canonical PBC containing protein families from the ones that diverge from this motif are shown in Figure 6a. Notably, nearly all the distinguishing residues are clustered around the cAMP binding site in the beta subdomain (Figure 6b). The only exception is G169, which is located in the β2-β3 loop (Figure 6a). Gly169 does not directly interact with cAMP, but still appears to be co-conserved with residues in the cAMP binding pocket. A careful analysis of the structural interactions associated with Gly169 indicates that the Cα of Gly169 mediates a CH-π interaction with the guanidium group of Arg209, which in turn coordinates with the phosphate group of cAMP (Figure 6b). Thus, although Gly169 does not directly interact with cAMP, it appears to be structurally linked to the phosphate group of cAMP via Arg209. Why would this structural link be important?

Recent NMR studies on the PKA regulatory subunit had suggested a key role for the β2-β3 loop in coupling cAMP signals to distal regulatory sites [18]. Specifically, the backbone amide of Gly169 was shown to undergo large chemical shift changes upon binding to cAMP. This change was proposed to alter the conformation of an adjacent aspartate (Asp170), the backbone of which forms an N-cap to the B/C-helix (Figure 6b). Because the B/C helix forms a docking site for the catalytic subunit, this coupling between the PBC and the B/C-helix (via the β2-β3 loop) was proposed to play a key role in PKA allostery [18]. The co-conservation of Gly169 with Arg209 suggests that this allosteric coupling may have specifically evolved in CBDs that bind to cAMP. Notably, MARR-bacteria and ASNC-bacteria (Figure 6a) are two families that conserve Arg209 in the PBC, but lack Gly169 in the β2-β3 loop. These two families presumably may have evolved alternative mechanisms of regulation. Future studies will focus on delineating these mechanisms using a combination of computational and experimental techniques.

## Conclusion

A global analysis of CNB domain containing sequences in the public and GOS data has provided novel insights into the evolution of CNB domain structure and function. Two evolutionary events appear to have contributed to CNB domain functional divergence, domain recombination and sequence variation. The sequence diversity observed within the PBC suggests that the CNB domain has evolved as a scaffold for not only binding cAMP, but also a wide variety of other ligands, many of which are yet to be characterized. Statistical comparison of the evolutionary constraints acting on the canonical PBC motif containing CNB domains with the non-canonical ones reveals that the residues in the PBC region have co-evolved with residues in the β2-β3 loop. Examining these constraints in light of structural and biochemical data provides a model of allosteric regulation, which is likely conserved in all cAMP binding modules. The results described in this study have implications for protein engineering and for the design of allosteric inhibitors.

## Materials and methods

### Identification of CNB domains

CNB domains in GOS and NR data were identified using a combination of psi-blast [29] and Gibbs motif sampling procedures [30]. Psi-blast profiles and motif models were initially built using CNB domains of known structures. These models were then iteratively updated as distant members from NR and GOS data were identified. An e-value cutoff of 0.001 was used for psi-blast searches.

### Classification of CNB domains in NR

CNB domains identified from NR (5,241 sequences) were multiply aligned using the CHAIN analysis program [28]. The aligned sequences were clustered into families and sub-families using the clustering option in the CHAIN program and the SECATOR program [31]. Families were annotated by identifying the functional domains linked to the CNB domain. The taxonomic origin of the sequences was also taken into account in the annotation processes. For instance, PKG-like CNB domains from parasitic organisms were annotated as 'PKG_parasites'. Functional domains were identified using rpsblast, which was run against a collection of conserved domains in CDD, Smart and Pfam [32] with an e-value cutoff of 0.0001.

### Classification of Global Ocean Sampling CNB domain containing proteins

Because CNB domains in the GOS data displayed significant sequence similarity to known CNB domains, they were assigned to one of the 30 families by running them against 30 family specific blast profiles. The taxonomic assignment for the GOS sequences was likewise done based on their similarity to known NR sequences [19]. Examination of the domain organization in individual families indicated that while the NR sequence contained both the CNB domain and functional domains, GOS sequences usually contained only the CNB domain. This presumably is due to the fragmentary nature of the GOS data. In any case, nearly all the CNB domain containing GOS sequences could be assigned to one of the 30 families based on the similarity within the CNB domain alone.

### Visualization of phylogenetic trees

In order to visually examine the evolutionary relationship between the identified sequences, we first constructed a phylogentic tree of all the 7,696 CNB sequences. The resulting tree, however, was very complex and hard to interpret. Therefore, we decided to take an alternative approach where we depicted each family by a consensus sequence. The 30 consensus sequences, corresponding to each of the 30 families, were generated from multiple alignments of individual families. The neighbor joining algorithm as implemented in the Molecular Evolutionary Genetics and Analysis (MEGA) program [33] was used for tree construction and visualization. Bootstrap test was done using default settings in MEGA.

### Measuring the evolutionary constraints imposed on CNB sequences

The evolutionary constraints imposed on CNB sequences were measured using the CHAIN program [28]. In brief, the CHAIN program identifies co-conserved residues that distinguish two related sets of sequences (foreground and background) by measuring the degree to which aligned residue positions in the foreground set are shifted away from the corresponding position in the background set. Residue positions that are shifted the most (indicated by red histograms above the alignment) contribute to the functional divergence of the foreground set from the background set. In the current study, all the CNB sequences that contain the canonical PBC motif constitute the foreground set, while the ones that lack the canonical motif constitute the background set.

The sequence identifiers for the sequences used in alignments Figures 2, 5b and 6a are: 94370018|PDZ_GEF-mouse; 93138731|K-channel-plant; 9857982|FixK-bacteria; 6759981|Fnr-bacteria; 15675445|ArcR-bacteria; 17989331|NnR-bacteria; 68552962|CBS-bacteria; 15673985|Flp-bacteria; 56419292|ARSR-bacteria; 1942960|PKA-mouse; 37964177|PKG-seahare; 68076807|PKA-parasite; 76609590|Epac-cattle; 68402320|HCN-zebrafish; 89309052|channel_Tetrahymena; 87198326|Bact_Pyrredox; 22298372|channel_Bact; 76259471|HisK-bacteria; 106879720|AAA_Atpase-bacteria; 462748|NtcA-bacteria; 86610079|ICLR-bacteria; 71367866|GNTR-bacteria; 111225891|CRP-bacteria; 115352640|MARR-bacteria; 116183754|ASNC-bacteria; 1FT9|pdb|cooA-bacteria; 2D93|pdb|PDZ_GEF_human; 2H6B|pdb|CprK-human.

## Abbreviations

CAP/CRP, catabolite activator protein; CBS, cystathionine beta synthase; CNB, cyclic nucleotide binding; GOS, Global Ocean Sampling; HisK, histidine kinase; HTH, helix-turn-helix; NR, National Center for Biotechnology Information's non-redundant amino acid database; PBC, phosphate binding cassette; PK, protein kinase.

## Authors' contributions

NK and SST conceived and designed the experiments. NK, JW performed the experiments. NK and SST analyzed the data. AFN, SY, GA and JCV contributed reagents/materials/analysis tools. NK and SST wrote the paper.
